# Colistin dry powder inhalation with the Twincer™: An effective and more patient friendly alternative to nebulization

**DOI:** 10.1371/journal.pone.0239658

**Published:** 2020-09-24

**Authors:** A. M. Akkerman-Nijland, F. Grasmeijer, H. A. M. Kerstjens, H. W. Frijlink, H. van der Vaart, J. M. Vonk, P. Hagedoorn, B. L. Rottier, G. H. Koppelman, O. W. Akkerman

**Affiliations:** 1 Department of Paediatric Pulmonology and Pediatric Allergology, Beatrix Childrens’ Hospital, University of Groningen, University Medical Center Groningen, Groningen, The Netherlands; 2 Groningen Research Institute for Asthma and COPD (GRIAC), University of Groningen, University Medical Center Groningen, Groningen, The Netherlands; 3 Department of Pharmaceutical Technology and Biopharmacy, University of Groningen, Groningen, The Netherlands; 4 PureIMS B.V., Roden, The Netherlands; 5 Department of Pulmonary Diseases and Tuberculosis, University of Groningen, University Medical Center Groningen, Groningen, The Netherlands; 6 Department of Epidemiology, University of Groningen, Groningen, The Netherlands; St. John's University, UNITED STATES

## Abstract

**Background:**

Nebulization of antimicrobial drugs such as tobramycin and colistin is a cornerstone in the treatment of patients with cystic fibrosis (CF) infected with *Pseudomonas aeruginosa*. However, nebulization has a high treatment burden. The Twincer^™^ is a dry powder inhaler specifically developed for the inhalation of antibiotics such as colistin. The aim of this study was to compare patient outcomes and experience with colistin dry powder by the Twincer with nebulization of colistin or tobramycin in adult CF patients in a real-life setting.

**Methods:**

This was a retrospective study from 01-01-2015 until 01-07-2018. Effectiveness was evaluated by comparing FEV_1_ decline and exacerbation rate during a mean of 4.1 years of nebulization therapy prior to the initiation of the Twincer against the same values during a mean of 1.7 years of treatment with the Twincer.

**Results:**

Twenty-one patients were evaluated, of whom twelve could be included in the effectiveness analysis, with a total of twenty patient years. Of all patients 71.4% preferred therapy with the Twincer over nebulization. Twincer use resulted in high treatment adherence with an average adherence rate of 92.5%. There was no significant difference in annual decline in FEV_1_%pred prior to and after start changing from nebulization to the use of the Twincer powder inhaler (median decline -1.56 [-5.57–5.31] and 1.35 [-8.45–6.36]) respectively, p = 0.45 (linear mixed effect model)). No significant difference was found in the number of intravenous or combined total intravenous and oral antibiotic courses during Twincer therapy compared to when using nebulization (1.68 and 2.49 courses during Twincer therapy versus 1.51 and 2.94 courses during nebulization, p = 0.88 and p = 0.63).

**Conclusion:**

Colistin dry powder inhalation with the Twincer is a more patient friendly alternative to nebulization, and we did not observe significant differences in the clinical outcome, regarding lung function and exacerbation rates.

## Background

Over the past decades, life expectancy of people with cystic fibrosis (CF) has significantly improved as a result of improved antimicrobial treatment, new therapies (such as the CFTR modulators), management strategies aimed at improving nutritional status and facilitating mucus clearance, and standardization of care in multidisciplinary CF centers [1, 2]. These advances in therapy come at a price of a significant increase in treatment burden: it requires multiple medical treatments on a daily basis, taking a patient approximately 2–3 hours each day [3].

One of the cornerstones in the treatment of patients with CF is the inhalation of drugs, mainly antimicrobial drugs against *Pseudomonas aeruginosa*. The antimicrobial drugs for inhalation currently available in Europe are tobramycin, colistin, aztreonam and levofloxacin. The best studied and most frequently applied method of administration for inhaled antibiotics is by wet nebulization. Nebulization however has several disadvantages. The administration time via nebulization is approximately 10–20 minutes per dose. Cleaning and sterilization of the device after each use is extra time consuming as well, with approximately 20 minutes per use [4, 5]. Furthermore, nebulization solution needs refrigeration and the use of nebulizers requires an external power source, thereby limiting mobility [6]. Other more technical disadvantages of nebulization are the risk of auto-re-infection [7], a low lung deposition and pollution of the surrounding environment with the nebulized drug [8, 9]. Many of these disadvantages cumulate into a high treatment burden [10]. High treatment burden in general leads to lower adherence, which may in turn lead to poorer health outcomes including more frequent exacerbations, more rapid disease progression and increased absence from work or school [11, 12].

Dry powder inhalers (DPIs) are an attractive alternative to nebulization as it overcomes many of the disadvantages of nebulization. First of all, DPIs have a much shorter administration time than nebulization, with a DPI taking approximately 1–2 minutes per dose [13]. Secondly, dry powders are in general more stable than solutions, thus eliminating the need for a cold chain transport and storage. Thirdly, DPIs do not require a power source, thereby increasing mobility. Finally, with an efficient DPI, a three to six-fold higher lung deposition can be obtained compared to a nebulizer [9, 14, 15].

Unfortunately, only a few dry powder inhalation systems are currently available that can handle the high doses required for pulmonary administration of antibiotics. The Twincer^™^ is a dry powder inhaler designed for the inhalation of colistimethate sodium (‘colistin’ in the remainder of this text) [16]. Though not registered, it has been available in the Netherlands in compassionate use programs since 2015. It is a disposable inhaler designed for single use and the delivery of large powder doses. Our hypothesis is that these advantages of this DPI increase patient satisfaction and adherence and therefore lead to improved clinical outcome.

The aim of this study is to evaluate the real-life clinical experience of the Twincer in adult CF patients, by describing its tolerability, patient satisfaction, adherence, clinical outcomes as lung function decline and exacerbation rate, and comparing this to nebulized antibiotics.

## Methods

### Study design and population

A retrospective study from 01-01-2015 until 01-07-2018 was conducted. The study included adult CF patients from the CF center of the University Medical Center Groningen (UMCG). All patients prescribed colistin from the Twincer on the basis of ‘medical need’ were included. This included patients experiencing side effects using wet nebulization or inhalation via other dry powder devices, such as the Podhaler^™^ or the Colobreathe^™^, but also if there was non-adherence to previous treatment due to high treatment burden. All patient data were extracted from hospital electronic patient files. The Medical Ethics Committee of the UMCG granted a waiver (METc 2018/488), as they concluded that this study was not subject to the Dutch Medical Research Involving Human Subjects Act (WMO).

### Patient experience and adherence

Information about patient experience was gathered through the distributing pharmacist. All patients were contacted monthly and asked about their experience with the Twincer in a semi-structured way, using questionnaires. They were asked for their opinion about the Twincer, the advantages and disadvantages, possible side effects and the inhalation “maneuver” itself (Table 1). Because only one pharmacy distributed this DPI, adherence could be evaluated as well.

**Table 1:** Questionnaire.

• What is your opinion about the Twincer?
• When you are satisfied with the Twincer, what is the most important advantage?
• When you are not satisfied with the Twincer, what is the most important disadvantage?
• Do you experience side effects?
○ Cough?
○ Bad taste?
○ Other?
• How is the inhalation via the Twincer?

### Clinical outcome

Clinical outcome consisted of FEV_1_ and the frequency of exacerbations. We compared the course of FEV_1_ and exacerbation rate in the 1–5 years of nebulization therapy prior to the initiation of treatment with the Twincer against these parameters during treatment with the Twincer. This was only possible for patients that had been using colistin or tobramycin nebulization before the Twincer. For FEV_1_ we used the best FEV_1_%predicted per quarter of the year. Exacerbations treated with an intravenous antibiotic course and exacerbations treated with oral antibiotics were recorded.

### Inspection of the DPI

The effectiveness of the inhalation maneuvers of the patients was evaluated by inspection of the discharge of the colistin from the used Twincers. To make this feasible, the used Twincers were collected by the pharmacy in sealed bags. After that, they were analyzed and scored with a discharge score to determine how much colistin remained in the device after inhalation. The discharge scores were classified as follows: 1 = very good discharge, classifiers exceptional clean; 2 = good discharge, minor amount of powder remaining in blister and/or classifiers; 3 = adequate discharge, a few agglomerates remained in blister and classifiers; 4 = moderate discharge, agglomerates in blister and/or bypass channel and/or above average powder in the classifiers; 5 = inadequate discharge, complete blockage of powder channel. The discharge scores were related to an emitted dose by the manufacturer of the Twincer during quality control. For quality control the Twincers were connected to a device generating 4 kPa of underpressure for three seconds to simulate an inhalation maneuver. The discharge score was then determined by the pharmacy assistant. Thereafter, the mass difference before and after the simulated inhalation maneuver was determined and regarded representative of the emitted colistin dose. Scoring of the used Twincers was always performed by the same pharmacy assistant. The discharge scores were compared with previous discharge scores of the same patient. If a deterioration was observed, inhalation instruction and training was offered again.

### Inhalation instruction and flow profiles

Before starting treatment with the Twincer, all patients received an inhalation instruction. The training consisted of a one-on-one physical demonstration with a dummy connected to a laptop on which their inhalation flow curve was shown in real time. The visual feedback from the flow curves was exceptionally valuable in teaching the patients a proper inhalation technique, as it enabled a clear, unambiguous instruction to the patient on the inspiratory effort required for a proper functioning of the inhaler. Prior to the start of the nebulizations, the patients were instructed as usual in clinical care, but did not receive any extensive training.

### Statistics

Descriptive statistical methods were used (mean, SD, median, range) for the entire study population. Mann-Whitney U and Wilcoxon signed rank testing were used to compare exacerbation rates. For evaluating mean annual decline in FEV_1_ linear mixed effect models were used. Following the methodology described by Naumova [17], time was defined as the time relative to the start of the use of the Twincer in years. Estimations of the annual FEV_1_ decline and FEV_1_ levels (i.e. intercepts) were made for the periods before and after the start of the Twincer by including the variables ‘time’, ‘treatment with the Twincer’ and the interaction between ‘time’ and ‘treatment with the Twincer’. Furthermore, individual variations in these declines and intercepts were accounted for by estimating the random effects for these variables.

## Results

In total twenty-one CF patients were included in this study. For twenty patients, colistin was prescribed as suppressing treatment for chronic *P*. *aeruginosa* infection. The other patient started with colistin for an *Achromobacter species* infection. Of the twenty-one patients, seven switched to the Twincer due to complaints using nebulization or other dry powders, six patients were non-adherent to nebulization therapy, six patients preferred starting with / switching to the Twincer because of concomitant use of other dry powder therapy, and two patients because they deteriorated using nebulization therapy. Before the switch to the Twincer patients used various nebulizers but did not change their nebulizer during the period studied. Baseline clinical characteristics are displayed in Table 2.

**Table 2:** Baseline characteristics.

	Total population (*n* = 21)	Effectiveness analyses (*n* = 12)
**Gender, *n* (%)**		
** **Male	13 (61.9)	8 (66.7)
** **Female	8 (38.1)	4 (33.3)
**Age in years, mean (range)**	29.2 (18.0–50.0)	32.8 (18.5–50.0)
**BMI, mean (range)**	21.0 (16.2–31.1)	22.3 (19.6–31.1)
**CFTR mutation, *n* (%)**		
** **Homozygote Phe508del	13 (61.9)	7 (58.3)
** **Heterozygote Phe508del	7 (33.3)	4 (33.3)
** **Other	1 (4.8)	1 (8.3)
**Comorbidities, *n* (%)**		
** **Cystic fibrosis-related	7 (33.3)	5 (41.7)
** **diabetes (CFRD)		
** **Cystic fibrosis-related liver	8 (38.1)	6 (50.0)
** **disease (CFLD)		
** **Pancreas insufficiency	19 (90.5)	11 (91.7)
** **Osteoporosis	6 (28.6)	2 (16.7)
**Forced Expiratory Volume in one second**		
** **Percentage of predicted,	52.6 (23–86, ±20.8)	54.6 (23–82, ±19.7)
** **mean (range, ±SD)		
** **Absolute, mean (range, ±SD)	1.99 (0.78–3.1, ±0.8)	2.03 (0.89–3.1, ±0.74)
**Azithromycin use**	20 (95.2)	12 (100.0)
**Duration Colistin nebulization**		
** **Days (range, ±SD)		1508 (562–1827, ±2504.2)
** **Years (range, ±SD)		4.1 (1.5–5.0, ±1.4)
Duration Twincer		
** **Days (range, ±SD)		610 (199–1055, ±302.1)
** **Years (range, ±SD)		1.7 (0.6–2.9, ±0.8)
**Mean no. of IVAB courses**		
** **Nebulisation (range, ±SD)		1.51 (0–3.81, ±1.35)
** **Twincer (range, ±SD)		1.67 (0–3.48, ±1.55)
**Mean no. of all AB courses**		
** **Nebulisation (range, ±SD)		2.70 (0–8.37, ±2.34)
** **Twincer (range, ±SD)		2.28 (0–6.15, ±1.93)

The data of twelve of the twenty-one patients could be used for effectiveness analyses, as these patients used nebulization therapy >1 year before start with the Twincer (nine patients used colistin nebulizations, one used tobramycin nebulizations, and 2 patients used alternating colistin and tobramycin nebulizations). The other nine patients could not be used for the effectiveness analyses for the following reasons: five patients (23.8%) discontinued the Twincer within two months of commencement, two patients (9.5%) used DPI tobramycin before switching to the Twincer, one patient (4.8%) did not have inhalation therapy before initiation with the Twincer and one patient (4.8%) started with colistin as treatment for an *Achromobacter species* infection.

### Patient experience

Five of the twenty-one patients (23.8%) discontinued the Twincer within 2 months of commencement. Three of them discontinued due to cough, one due to dyspnea/bronchospasms and one due to throat discomfort. Four out of these five patients had previously also been intolerant of colistin nebulization therapy due to cough (2), throat discomfort (2), bronchospasm (1) and hoarseness (1). The fifth patient had previously been on tobramycin nebulization therapy with complaints of cough. After discontinuation of the Twincer none of the patients returned to colistin nebulization; three patients returned to tobramycin nebulization (two of which switched to levofloxacin nebulization in the next couple of months), one patient switched to dry powder tobramycin and the last one stopped with all inhalation therapy. Two patients discontinued the Twincer after approximately one year of therapy. One patient discontinued due to regular complaints of cough and restarted with colistin nebulizations in the months before lung transplantation. The other patient wanted to try colistin nebulization therapy because of incidental cough but switched back to the Twincer after a few months of nebulization due to convenience of the administration by the Twincer.

Of the total population of twenty-one cystic fibrosis patients, three patients reported never having complaints of cough (14.3%), eight patients (38.1%) rarely had complaints of cough, six patients (28.5%) reported sometimes having complaints of cough, and four patients (19.0%) regularly complained of cough. Four patients that reported incidental or regular cough, stated that when they inhaled more slowly, cough was diminished. Three patients reported a bad taste after inhalation of colistin (Table 3).

**Table 3:** Side effects.

Side effects	Continuation Twincer	Discontinuation Twincer
	16 / 21	5 / 21
	(76.2%)	(23.8%)
**Cough**		
• Total	13	5
• Rarely / incidental	6	2
• Sometimes	6	-
• Regularly	1	3
**Dyspnea/bronchospasm**	-	1
**Throat discomfort**	-	1
**Bad taste**	1	2

Sixteen of the twenty-one patients (76.2%) were satisfied to very satisfied with the Twincer. Of these, twelve patients could compare the dry powder colistin to colistin nebulization. All of them preferred the Twincer over nebulization, with ‘convenience’ and ‘time saving’ as the most common reasons. However, one of the patients eventually switched back to nebulizations after about one year, despite earlier preference for the Twincer, due to his deteriorating health condition with increasing complaints of dyspnea.

### Adherence

Based on distribution data from the pharmacy, adherence rates were found between 66.0% and 100% when using the Twincer, with an average adherence rate of 92.5% (median 99.5%).

### Effectiveness

The data of twelve of the twenty-one patients could be used for effectiveness analyses, as these patients used nebulization therapy 1–5 years before start with the Twincer (nine patients used colistin nebulizations every other month, one used tobramycin nebulizations every other month, and 2 patients used alternating colistin and tobramycin nebulizations). These twelve patients have used the Twincer for a total of 20 patient years in comparison to 49.6 patient years of wet nebulization. Data from the other nine patients could not be used for the effectiveness analyses for the following reasons: five patients (23.8%) discontinued the Twincer within two months of commencement, two patients (9.5%) used DPI tobramycin before switching to the Twincer, one patient (4.8%) did not have inhalation therapy before initiation with the Twincer and one patient (4.8%) started with colistin as treatment for an *Achromobacter species* infection.

### Lung function

Mean annual decline in FEV_1_ during nebulization therapy was 1.1% predicted (median -1.56 [-5.57–5.31]). After start of the Twincer annual decline in FEV_1_ was 2.2% predicted (median 1.35 [-8.45–6.36]). Linear mixed effect models showed no significant difference in mean annual decline in FEV_1_ prior to and after start of the Twincer (p = 0.45).

**Fig 1:**
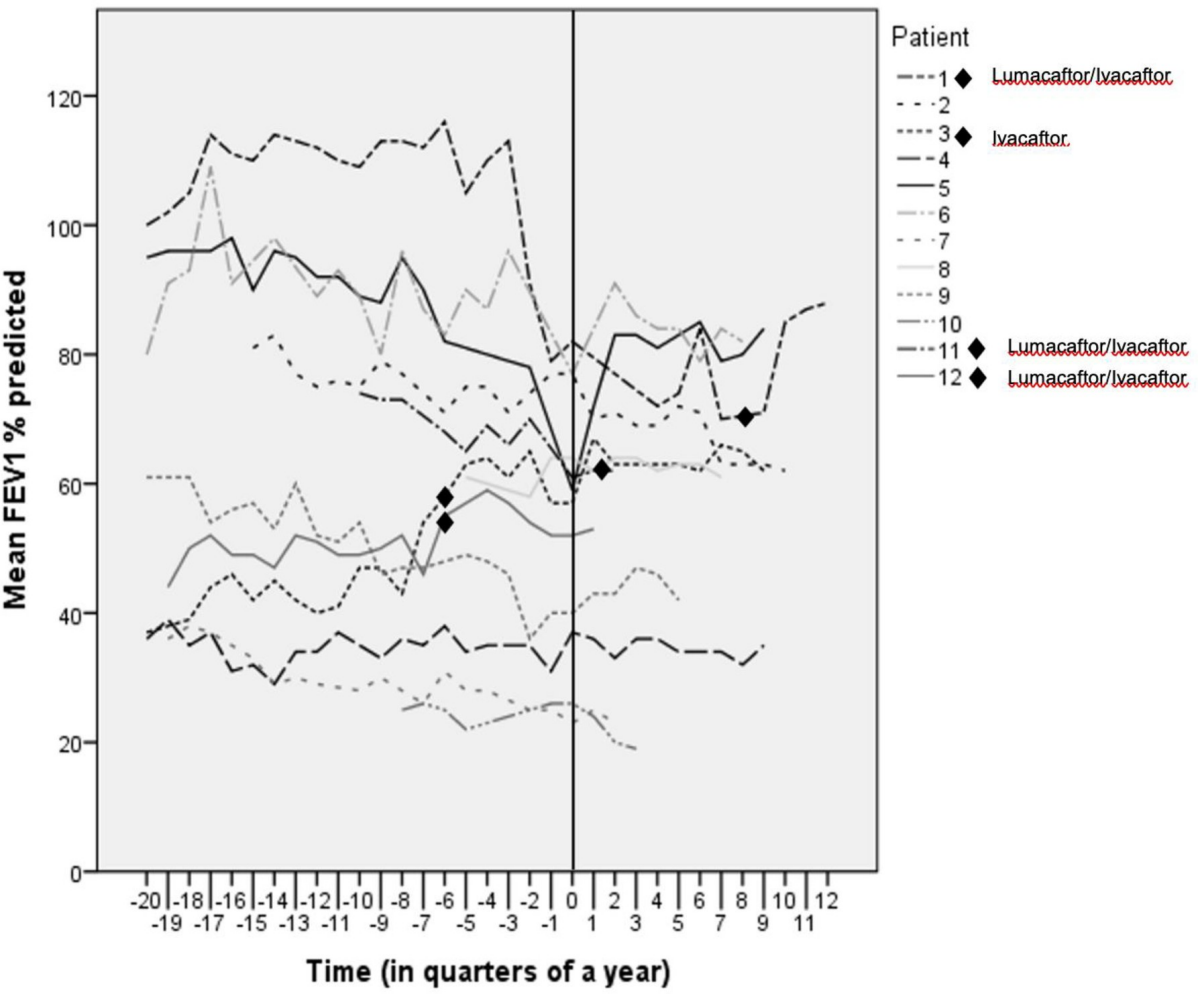
Course of the FEV_1_ in % predicted for all 12 patients. Time = 0 means start of the Twincer. One of the patients who started with lumacaftor/ivacaftor and the patient on ivacaftor started 1,5 years before start of the Twincer. One patient on lumacaftor/ivacaftor started around the same time as he started with the Twincer, and the last patient on lumacaftor/ivacaftor started 2 years after start of the Twincer.

Fig 1 shows the course of FEV_1_ in % predicted for all twelve patients. A complicating factor in the analysis of the effectiveness of the Twincer is the fact that during the study period four of the twelve patients also started with the CFTR modulators lumacaftor/ivacaftor (three patients) or ivacaftor (one patient), of which the starting point is indicated in Fig 1. This can have an effect on the analysis as CFTR modulator therapy can lead to an improvement in lung function and a reduction in the number of exacerbations among other things. When eliminating these four patients, linear mixed effect models showed a mean annual decline in FEV_1_ during nebulization therapy of 2.5% and of 1.1% when using the Twincer^™^ (p = 0.19).

### Exacerbation rate

The mean number of intravenous antibiotic courses was 1.51 per year during nebulization versus 1.67 per year during treatment with the Twincer. Mean number of all antibiotic courses (intravenous and oral courses combined) was 2.70 courses per year for the nebulization period and 2.28 courses per year during Twincer therapy. There are no significant differences between the groups (p = 0.88 and p = 0.63).

### Inspection of the used and returned Twincers

Between 7 October 2015 and 6 June 2018 in total 1036 Twincers of various produced batches of Twincers were used for quality control, including in vitro dispersion measurements for evaluating the discharge score. Mean discharge dose per discharge score at quality control are shown in Table 4.

**Table 4:** Mean emitted dose per discharge score at quality control (n = 1036), and frequency per discharge score of all used and returned Twincers (total n = 8544; for discharge score 2–5 than n = 6963).

Discharge score	N in quality control	Mean emitted dose in mg (SD)	Frequency in the 8544 returned Twincers in %
**2**	202	50.9 (1.5)	14.6
**3**	671	44.2 (3.5)	56.7
**4**	147	36.3 (5.8)	9.7
**5**	16	19.6 (10.4)	0.5

The discharge scores were classified as follows: 1 = very good discharge, classifiers exceptional clean; 2 = good discharge, light smearing in blister and/or classifiers; 3 = adequate discharge, moderate smearing of little agglomerates in blister and classifiers; 4 = moderate discharge, agglomerates in blister and/or bypass channel and/or above average smearing of classifiers; 5 = inadequate discharge, complete blockage of powder channel.

A discharge score of ‘1’ was never found during in vitro quality checks. Although the determination of the discharge score is somewhat subjective, a discharge score of ‘1’ of the used and retrieved Twincers often will be the consequence of poorly sealed packaging (minigrip bag) and subsequent liquefaction of the powder by hygroscopy. This score is, therefore, considered an artefact.

In total 8544 used Twincers of the included patients were retrieved and scored with the aforementioned discharge score. The majority of the scored Twincers had a discharge score of adequate to very good (in total 89.8%; Table 4). This means that 89.8% of the used Twincers emitted a dose between 44.2–50.9 mg. Only 0.5% had an inadequate or very poor discharge score and 9.7% a moderate or poor discharge.

## Discussion

This study describes a total of 20 patient years of experience of colistin inhalation using the Twincer dry powder inhaler, assessing its patient satisfaction, tolerability, adherence, clinical outcomes, and comparing this to nebulization. Overall, of the twenty-one patients who started with the Twincer (five discontinued the Twincer within two months of commencement due to side effects) sixteen tolerated the Twincer well with high patient satisfaction and patient preference. Despite a preference of the Twincer, one patient switched back to colistin nebulizations in the few months before lung transplantation. The most important benefits of Twincer use mentioned were ‘convenience’ and ‘time-saving’. We did not observe significant differences during treatment with the Twincer and nebulization therapy regarding the frequency of exacerbations and course in FEV_1_.

Our study shows a high patient satisfaction with the Twincer. Sixteen out of twenty-one patients (76.2%) were satisfied to very satisfied with the device. Most reported satisfying features are ease of use, fast and tolerable administration, and portability. Other studies have also found higher preferences for DPI over nebulization. In the Freedom study 65.6% of 183 patients preferred treatment with DPI colistin over nebulization therapy with tobramycin, which they had been using pre-randomisation [18]. In a real-life clinical experience of Harrison, 96% of patients who had previously used tobramycin nebulization preferred DPI tobramycin therapy over nebulisation [5]. Although DPI treatment seems to be generally preferred over nebulization, patient satisfaction will depend on the specific type of dry powder inhaler used. Moreover, in our study we did not randomize subjects to nebulization versus DPI but describe the real-world outcome of the therapy in our patients. It is possible that patients who prefer nebulization did not agree to try DPI.

Within the entire patient population of this study, cough was found to be the most common adverse event, with 85.7% of patients indicating they at least rarely experienced cough. Four patients who sometimes or regularly perceived side effects of cough stated that when they inhaled more slowly cough was diminished. Because the delivered dose from the Twincer inhaler was not found to decrease upon a slower inhalation in *in vitro* testing, this is an effective way to reduce side-effects. The success of this approach can be ascribed to a lower and less forceful throat and upper airway deposition. In the Freedom study cough was also the most common adverse event, occurring in 75.4% of colistimethate sodium DPI recipients (total of 186 patients; via the Colobreathe) versus 43.5% of nebulized tobramycin recipients (total of 193 patients). They also reported a higher incidence of abnormal taste (62.6% vs 27.5%) and throat irritation (45.5% vs 28.0%) in the colistemethate sodium DPI group. Dyspnea was found in similar rates between both groups (26.2% vs 26.9%) [18]. A clinical comparison of DPI colistin (Colobreathe) and nebulized colistin also reported a higher incidence of cough (81.3% in 16 patients vs 46.7% in 15 patients) and throat irritation (81.3% vs 20.0%) in the DPI group [19]. Harrison et al found no significant differences in reported cough score between participants of the nebulization group compared to the DPI group [5].

The five patients that discontinued treatment with the Twincer within two months had previously also been intolerant of colistin/tobramycin nebulization therapy because of cough or dyspnea, indicating that the pharmacological agent is a relevant factor contributing to cough and other side-effects. One of the patients eventually switched back to nebulization therapy after about one year, despite earlier preference for the Twincer. Another patient wanted to try colistin nebulization but switched back to the Twincer after a few months of nebulization therapy. The EAGER trial reported a lower discontinuation rate with tobramycin inhalation powder due to adverse effects (13.0%; 40/308), than found in our study. Their overall discontinuation rate was, however, 26.9% [4]. In the study of Harrison et al 14% (10/73) discontinued TIP within one month of commencement. The reasons for this were cough or bronchospasm (9/10), one patient discontinued due to hemoptysis [5].

In general, cough, throat irritation and unpleasant taste are known side-effects of dry powder therapies, not only in DPI colistin but also in DPI tobramycin, due to deposition in the oropharynx [4]. It is thought that cough generally reduces over time with improved technique, and to some extent may be controlled by the use of bronchodilators in some patients [19].

We observed high adherence to dry powder inhalation using the Twincer, with an average adherence of 92.5% and a median of 99.5%. This high adherence is probably the consequence of intensive supervision by the pharmacist. Unfortunately, we do not have information about the adherence during nebulization therapy. Literature shows adherence rates during nebulization therapy around 65–80% via self-reporting, 50–60% via clinician reporting and 36% via electronic download [20], indicating a much higher adherence rate of Twincer therapy.

Harrison et al reported a significant improvement in self-reported adherence scores, with almost doubling the proportion of excellent adherence after transition to DPI tobramycin (43% to 83%) [5]. The importance of adherence is demonstrated by Briesacher, who related a higher adherence of inhalation therapy (nebulization) to a decreased risk of hospitalizations [21]. In our study, the periodic follow-up is not totally representative of clinical practice and may have led to higher adherence. On the other hand, our follow-up was not comparable to a trial setting. Moreover, a program with proper inhalation instructions and good follow-up by a distributing pharmacist might be feasible and even desirable on a larger scale.

In spite of the fact that the dose administered via the Twincer was only one third of the dose administered via the nebulizer (55 mg vs 160 mg; due to the higher lung deposition with less contamination of the surrounding environment), no significant difference was found in the rate of decline in FEV_1_. The lack of a significant difference in rate of decline in FEV_1_ between antibiotic nebulization and dry powder antibiotics is in agreement with data from previous studies [5, 18]. We did, however, observe significant variation in rate of lung function decline between patients before and after start of the DPI. Especially in the light of this considerable variability, a larger RCT is necessary to study this aspect further.

Exacerbation rates of dry powder inhalation and nebulization therapy appears to be similar in our study. This will not be generally applicable to other DPI and nebulization therapies, as this may strongly depend on the type of devices used and the antibiotic doses administered. For example, Schuster et al compared DPI colistin with nebulized tobramycin and found that more patients in the DPI colistin group experienced exacerbations compared to patients in the tobramycin nebulization group (31.1% vs 26.1%) [18]. In contrast, Harrison et al found a significant decrease in the number of intravenous antibiotic courses during 12 months of DPI tobramycin (75% of the participants required no intravenous antibiotics during DPI therapy compared with 44% of participants on nebulization therapy). They showed no significant difference in oral antibiotic usage [5]. In another comparison of DPI tobramycin and nebulized tobramycin, the EAGER trial reported similar number of hospitalizations for respiratory-related events for the tobramycin DPI and nebulization group (24.4% vs 22.0%) [4]. There are no other studies comparing DPI colistin to nebulized colistin.

Our study has a number of limitations. The most important limitation of the study is the small population size and the single-center design, limiting power and generalizability of the results. Furthermore, a selection bias might have occurred, as the Twincer was only prescribed to patients based on ‘medical need’. Besides this, data on patient preference were only available of patients who continued with the Twincer, not of the patients who stopped with the Twincer within two months of commencement but had previously also been intolerant of nebulization therapy. A disadvantage of retrospective real-life studies is that complete data collection is not guaranteed. Most of the information we know about inhalation therapy is derived from studies with a randomized clinical trial design. In that setting conditions are optimal, excluding many of the real-life factors that affect treatment efficacy and adherence. This study explored the effectiveness of treatment and adherence of patients during their everyday life. An example of incomplete data collection is the missing data on compliance during nebulization therapy, which limited a good comparison. However, we did have a good method of evaluating the adherence of DPI therapy, as there was only one distributing pharmacy and the used Twincers were returned and inspected.

## Conclusion

In conclusion, our real-life evaluation study, with a small number of patients, shows that colistin dry powder inhalation using the Twincer is a patient friendly alternative to nebulization, with no significant differences found in the clinical outcome regarding lung function decline and exacerbation rates.
